# Enabling SENSE accelerated 2D CSI for hyperpolarized carbon-13 imaging

**DOI:** 10.1038/s41598-024-70892-8

**Published:** 2024-09-04

**Authors:** Ayaka Shinozaki, Juan D. Sanchez-Heredia, Markus P. Andersen, Mohsen Redda, Duy A. Dang, Esben S. S. Hansen, Rolf F. Schulte, Christoffer Laustsen, Damian J. Tyler, James T. Grist

**Affiliations:** 1 Oxford Centre for Clinical Magnetic Resonance Research, University of Oxford, Oxford, UK; 2 Department of Physiology, Anatomy, and Genetics, University of Oxford, Oxford, UK; 3 JD Coils, Hamburg, Germany; 4 Department of Clinical Medicine, Aarhus University, Aarhus, Denmark; 5 GE HealthCare, Munich, Germany; 6 Department of Radiology, Oxford University Hospitals, Oxford, UK

**Keywords:** Magnetic resonance imaging, Medical imaging

## Abstract

As hyperpolarized (HP) carbon-13 (^13^C) metabolic imaging is clinically translated, there is a need for easy-to-implement, fast, and robust imaging techniques. However, achieving high temporal resolution without decreasing spatial and/or spectral resolution, whilst maintaining the usability of the imaging sequence is challenging. Therefore, this study looked to accelerate HP ^13^C MRI by combining a well-established and robust sequence called two-dimensional Chemical Shift Imaging (2D CSI) with prospective under sampling and SENSitivity Encoding (SENSE) reconstruction. Due to the low natural abundance of ^13^C, the sensitivity maps cannot be pre-acquired for the reconstruction. As such, the implementation of sodium (^23^Na) sensitivity maps for SENSE reconstructed ^13^C CSI was demonstrated in a phantom and in vivo in the pig kidney. Results showed that SENSE reconstruction using ^23^Na sensitivity maps corrected aliased images with a four-fold acceleration. With high temporal resolution, the kidney spectra produced a detailed metabolic arrival and decay curve, useful for further metabolite kinetic modelling or denoising. Metabolic ratio maps were produced in three pigs demonstrating the technique’s ability for repeat metabolic measurements. In cases with unknown metabolite spectra or limited HP MRI specialist knowledge, this robust acceleration method ensures comprehensive capture of metabolic signals, mitigating the risk of missing spectral data.

## Introduction

**Fig. 1:**
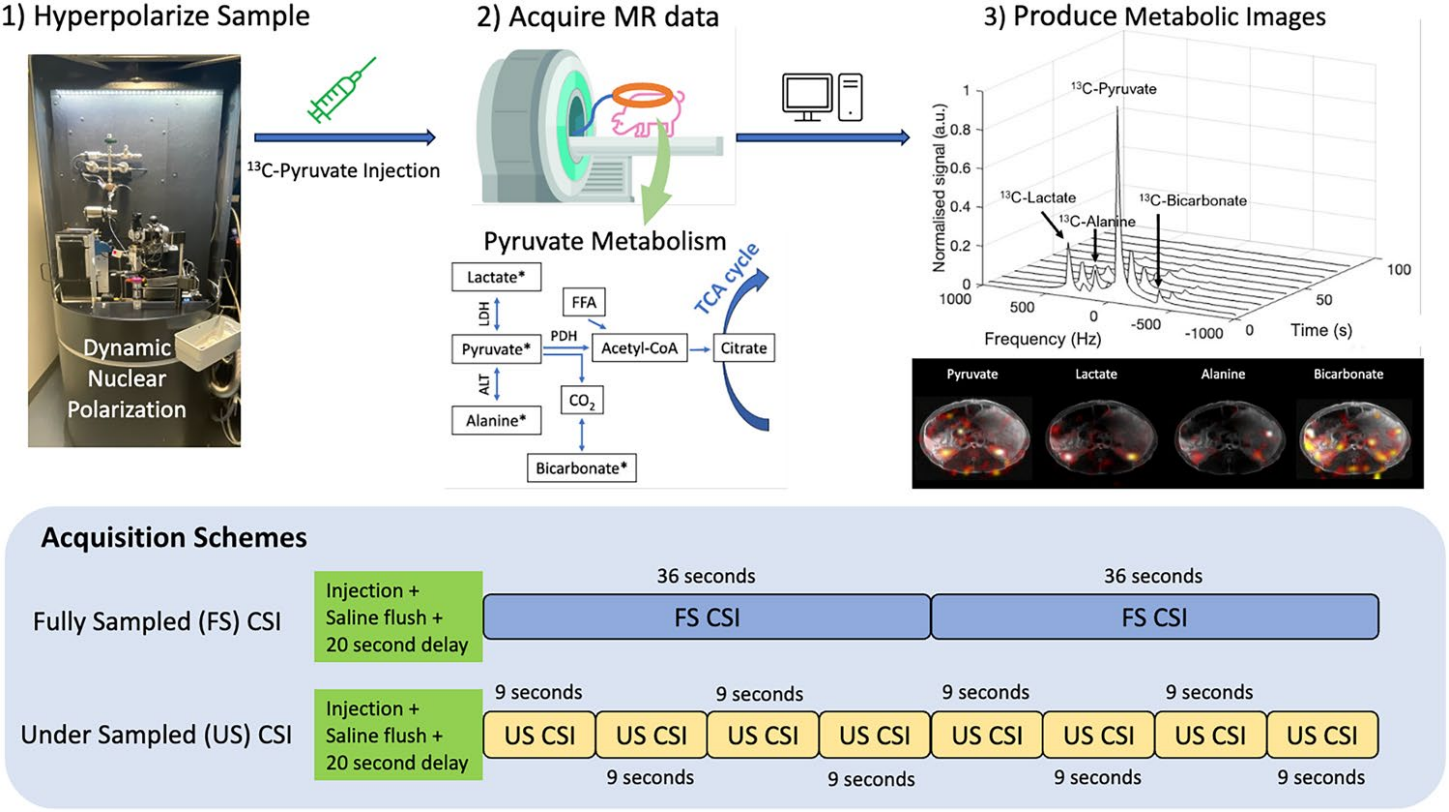
Overview of the HP ^13^C experimental work flow (steps 1–3) carried out in this study. The illustrations of the two CSI acquisition schemes are both FID CSIs taken consecutively following an injection, saline flush, and a 20 second delay. The fully sampled (FS) CSI (36 seconds per acquisition) and the under sampled (US) CSI (9 s per acquisition) are collected after separate HP ^13^C injections about 90min apart. To visualize the four-fold acceleration, the schemes are illustrated directly on top of each other, where four consecutive US CSIs are taken within a single FS CSI.

HP ^13^C MRI is a translational, clinical, non-invasive technique used to observe various metabolic pathways *in vivo* by injecting hyperpolarized ^13^C-labelled endogenous substrates^1^. The technique has the potential to provide key information to help further the understanding of early metabolic changes in pathology. Endogenous ^13^C is of low natural abundance (1% of all Carbon) and concentration (on the order of mM), thus has a low sensitivity^2^. To overcome the low *in vivo* signal-to-noise ratio (SNR), the signal from ^13^C-labelled endogenous substrates, such as [1-^13^C]pyruvate, can be enhanced via dynamic nuclear polarization (DNP), a process leading to a>10,000-fold increase in the signal from the ^13^C nucleus^3,4^, which is depicted in Fig. 1, step 1. The HP sample is injected into the subject and this allows for in vivo metabolism to be tracked through the acquisition of MR data as shown in Fig. 1, step 2. The data is then post-processed to produce metabolic images, shown in Fig. 1, step 3.

For [1-^13^C]pyruvate, one of the challenges is that the observation time in vivo is approximately two minutes before the signal returns to thermal equilibrium due to the rapid polarization decay of the HP injection^5^. To overcome this inherent time limitation, HP MRI requires fast imaging approaches^6^. However, obtaining higher temporal resolution necessitates careful optimization of trade-offs between: (1) accelerating data acquisition to capture dynamic metabolic events before the signal decays, (2) maintaining a high SNR for image quality, and (3) preserving spatial and/or spectral resolution for anatomical and functional detail. Another challenge is the need for RF efficiency as each RF pulse will irreversibly use up the hyperpolarized magnetization^7^. Therefore, small numbers of low flip angle pulses enable the hyperpolarized magnetization to be maintained for as long as possible, whilst a 90 degree pulse will use up all the hyperpolarized magnetization in a single acquisition.

Several approaches have been developed for fast HP ^13^C imaging. One solution is using spectral-spatial imaging (SPSP), where a metabolite-specific RF pulse excites a particular frequency combined with a fast imaging readout^8^ such as single-shot echo-planar^9^ or spiral imaging^10^. While SPSP offers high RF efficiency, its application hinges on prior knowledge of the target metabolites’ center frequencies and spectral characteristics^7^. Acquiring such information presents a significant challenge. Pre-scans, a standard approach for setting the center frequency, are not feasible before the injection of the HP ^13^C substrate. Consequently, an incorrect center frequency selection can lead to excitation of an unintended resonance, rendering the experiment unsuccessful^7^.

In fast spectroscopic imaging, echo-planar type gradients are used to increase efficiency of the acquisition and reduce frequency-encodes during k-space traversals such as EPSI, spiral, and radial^7,11–13^. These techniques have high temporal resolution but a limited spectral bandwidth and spatial resolution due to hardware slew rate limits^7^. If the spectral profile is well defined, multi-echo balanced steady state free precession (bSSFP) can be used, where data is acquired at echo times centered between the RF pulses^14^. However, the interpretation of metabolic data from these approaches is complex due to the nature of the bSSFP signal equation necessitating prior knowledge of the $\textrm{T}_1$ and $\textrm{T}_2$ of each metabolite. Another approach is to use Iterative Decomposition of water and fat with Echo Asymmetry and Least-squares estimation (IDEAL) encoding with single-shot readouts, providing some flexibility in centre frequency mis-estimation^15^. IDEAL has high temporal resolution but suffers from spectral bleed through artifacts^15^.

Alternatively, phase-encoded CSI is a robust multi-voxel imaging technique, which is available on all major scanner platforms and can be used to capture signal from a broad range of metabolites^16,17^. Due to its high spectral resolution, CSI does not require prior knowledge of the metabolite spectra and is insensitive to inhomogeneous magnetic fields. However, it is an RF-intensive, slow acquisition method requiring acceleration. Parallel imaging has become a standard approach for accelerating imaging and harnesses additional information about the placement and sensitivities of receiver coils to help spatially locate the acquired MR signal^18^. This approach can be used with CSI to reduce the number of phase-encoding steps required, which results in shorter imaging times and fewer RF excitations^19^.

There are multiple methods for parallel imaging including SENSitivity Encoding (SENSE)^20^, where image space acquisition is accelerated, resulting in a reduced field of view (FOV), and GeneRalized Auto calibrating Partially Parallel Acquisitions (GRAPPA)^21^, where k-space sampling is reduced. Although there are various methods to decrease k-space sampling^17,22–24^ and thus increase temporal resolution, limiting FOV is a simple implementation where image resolution is preserved. Therefore, SENSE was chosen for CSI acceleration in this work to further increase temporal resolution while preserving the broad-spectral information. However, one challenge is that SENSE requires the acquisition of the RF coil sensitivity profiles for reconstruction, but due to the low natural abundance of ^13^C, this is not possible to pre-acquire^23^. Multiple ways of estimating a coil profile for ^13^C have been shown, such as: auto-calibration^19,25^, which is obtained from the center of a fully sampled k-space acquisition; calibrationless^26^, which iteratively solves a matrix completion problem instead of acquiring a coil sensitivity map; and hardware approaches^10^, which allows for the acquisition of a spatially similar sensitivity map using a surrogate nuclei. Previous work has implemented a hardware solution whereby a dual-tuned sodium-carbon receive array enabled imaging with SPSP 3D blipped stack of spirals to successfully image a pig kidney and a human brain^10^. Sanchez et al. has shown that this sodium-carbon Rx coil obtains accurate ^23^Na sensitivity profiles, which can be used for the estimation of ^13^C sensitivity profiles at 3T^10^.

Therefore, the aim of this work was to accelerate CSI by limiting FOV and using the sodium-carbon coil mentioned above to increase temporal resolution whilst retaining spatial and spectral resolution. The receive surface coil used in this experiment is specifically designed such that the SNR is optimized for the ^13^C frequency and the coil coupling coefficients are designed to be the same at the ^13^C and ^23^Na frequencies using a tailored coupling matrix approach. As such, the sensitivity profiles are expected to be the same, as shown by Sanchez et. al^10^. Building on the hardware developments, the aforementioned coil was expected to correct for folded artifacts called aliasing, which is a characteristic of accelerated parallel imaging.

## Results

### Phantom study

**Fig. 2:**
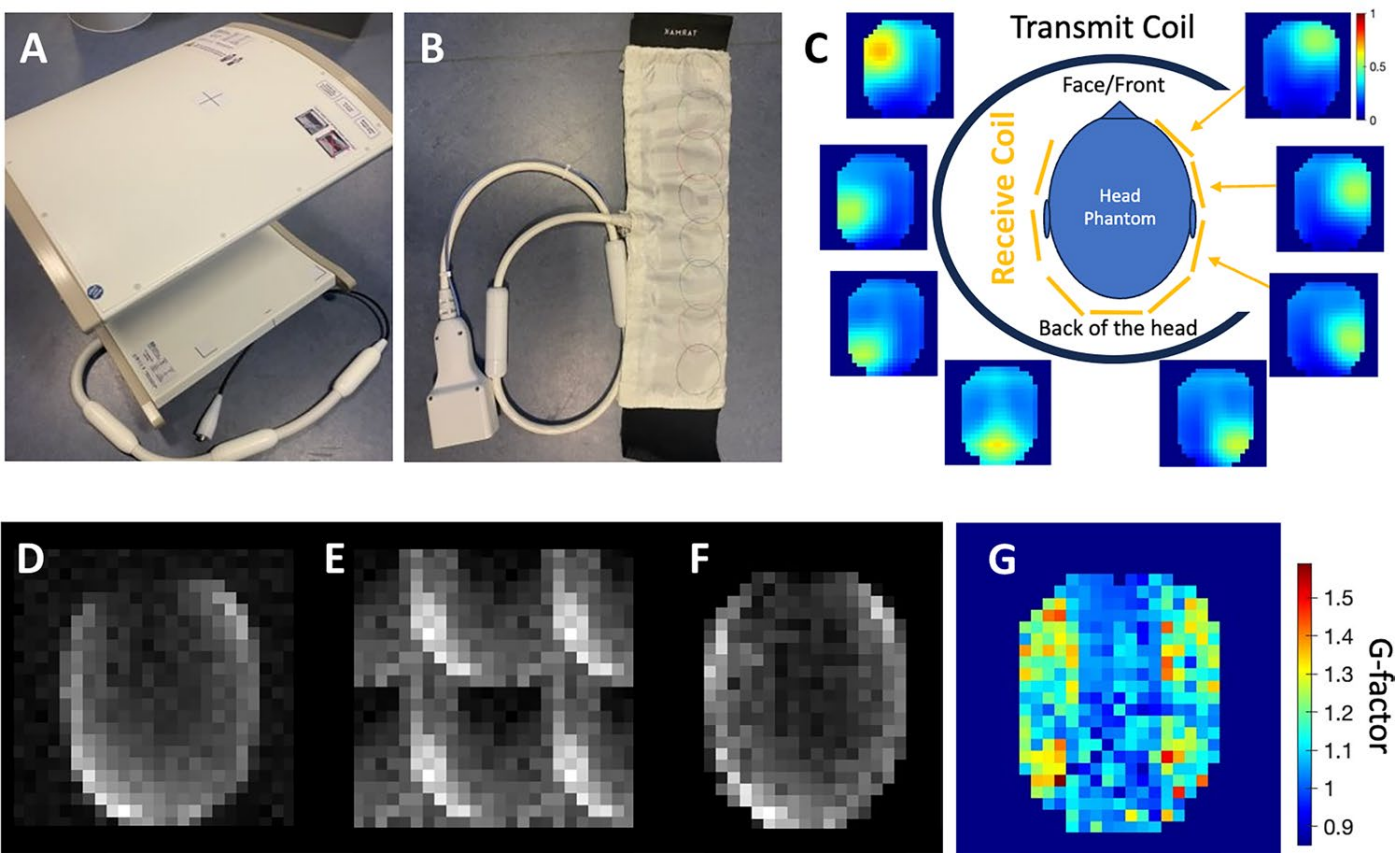
(**A**) ^13^C clamshell transmit coil (Rapid Biomedical, Germany) (**B**) an eight-channel array, flexible ^23^Na/^13^C receive coil (fabricated by Juan D. Sanchez-Heredia) (**C**) a phantom experiment set up is illustrated, where the head phantom sits inside the clamshell transmit coil and the head is wrapped with the receive coil. The location of the receive elements are depicted as yellow lines. Coil-wise sodium sensitivity images surround the illustration. (**D**) The fully sampled ^13^C phantom image. (**E**) The retrospectively under sampled ^13^C phantom image. (**F**) The under sampled SENSE reconstructed image. (**G**) The g-factor map of R = 4, where acquisition is accelerated by R = 2 in both the frequency and phase directions.

The viability of using sodium sensitivity maps for SENSE reconstruction of under sampled ^13^C images acquired from a Specific Anthropomorphic Mannequin (SAM) phantom is summarized in Fig. 2. An illustration of the experimental set up of a ^13^C clamshell transmit coil (Fig. 2A), a ^23^Na/^13^C receive coil (Fig. 2B), and a head phantom is shown in Figure 2C surrounded by the localized ^23^Na sensitivities of the eight receiver elements. Sensitivity drops rapidly away from the coil elements. As the loop coil was too short to wrap entirely around the phantom, the sensitivity profile has a “blind spot” at the top, where there are no elements as illustrated in Fig. 2C. Although the phantom was too large for the receive coil, this was not an issue for the reconstruction of images from this phantom nor with the subsequent in-vivo study.

The conventional, fully sampled CSI (Fig. 2D) shows signals acquired at the carbon frequency. Signal decreases toward the center of the phantom and reflects the “blind spot” at the top of the arc in Fig. 2D. The retrospectively under sampled CSI in Fig. 2E demonstrates the “wrap around” artifact characteristic of accelerated parallel imaging, where the anatomy is replicated and overlapped on the original structure. Figure 2F is the SENSE reconstructed under sampled image, where the aliasing is corrected through the use of the sodium sensitivity maps. The oval shape of the phantom, the opening at the top, and the gradual signal decrease toward the center of the phantom is recovered (Fig. 2F). The g-factor map is demonstrated in Fig. 2G, where the yellow-red regions on the upper left and upper right hand sides of the phantom highlight the noise amplification due to the coil hardware. The sodium sensitivity SENSE reconstruction (2F) recovered an image very similar to that of the fully sampled CSI (Fig. 2D) and this approach was subsequently implemented in-vivo.

### Porcine study

#### Sodium imaging

**Fig. 3:**
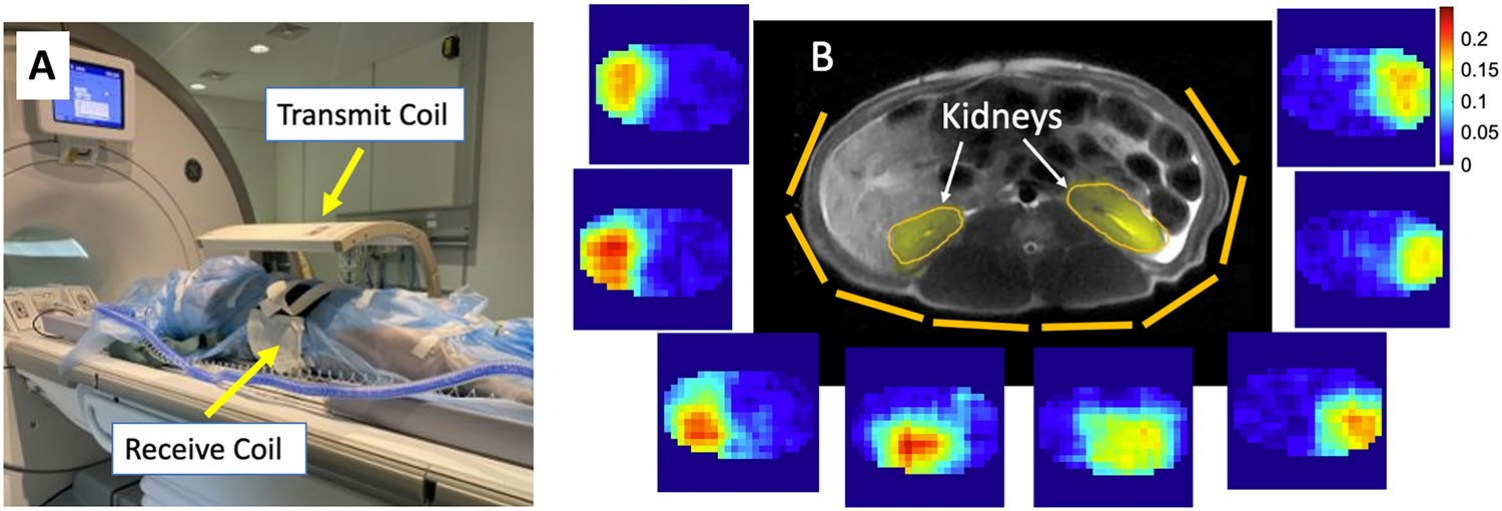
(**A**) A porcine experiment set up with a supine pig inside the clamshell transmit coil and the torso wrapped with the flexible receive coil. (**B**) Axial proton image of the porcine torso shows the location of the kidneys. The yellow lines visualize the flexible receive coil element positions and coil-wise sodium sensitivity images surround the proton image.

Figure 3A shows the porcine experimental set up within the MRI. In Fig. 3B, the torso anatomy of a representative pig is shown in an axial $\textrm{T}_2$ proton image, where [1-^13^C]pyruvate was expected to localise at the yellow kidney ROIs. Surrounding the proton image in Fig. 3B are the sodium sensitivity profile images of each receiver element, which were used as sensitivity maps for the subsequent SENSE reconstruction.

#### Carbon imaging

**Fig. 4:**
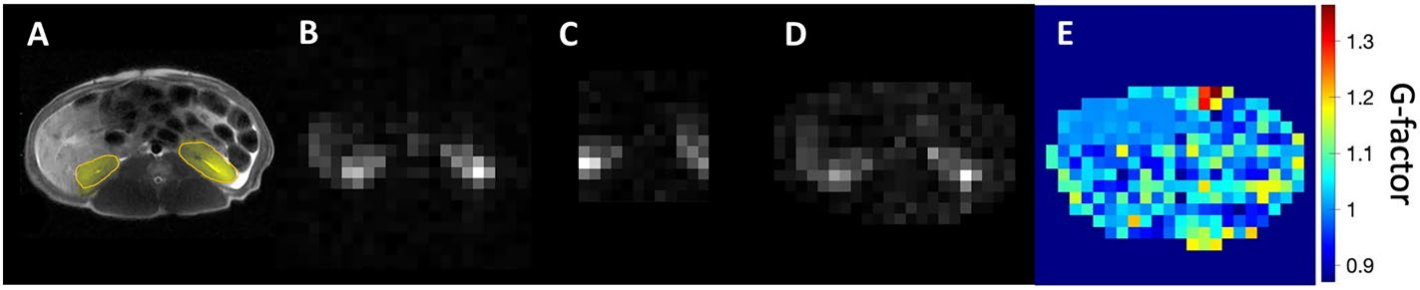
(**A**) Axial $\textrm{T}_2$ proton image of the pig anatomy with the kidneys highlighted in yellow. (**B**) [^13^C -1] pyruvate signal from fully sampled CSI taken over 36 seconds. (**C**) [^13^C -1] Pyruvate signal from under sampled CSI taken over 9 seconds. (**D**) [^13^C -1] pyruvate SENSE Reconstructed CSI based on the under sampled CSI and the sodium sensitivity image. (**E**) The g-factor map of R = 4, where acquisition is accelerated by R = 2 in both the frequency and phase directions.

Figure 4 demonstrates the performance of the SENSE reconstruction by comparing the pyruvate images produced through the conventional fully sampled CSI and the accelerated CSI methods. Figure 4A is the axial $\textrm{T}_2$ proton image with the highlighted kidney ROIs, where pyruvate was expected to localise. Figure 4B is the pyruvate signal from the fully sampled CSI showing two high intensity regions corresponding to the kidneys. Figure 4C demonstrates the aliasing due to the reduced FOV in the under sampled CSI. Figure 4D is the SENSE reconstruction of the under sampled image and two bright regions at the kidneys are recovered through the use of the ^23^Na sensitivity maps, illustrating the structural similarity between the fully sampled image (Fig. 4B) and the corrected under sampled image, which removes the aliasing (Fig. 4D). In Fig. 4E, the g-factor map quantifies the coil performance, where the highest values are localised at the top of the torso and away from the kidneys.

#### Spectral time course

**Fig. 5:**
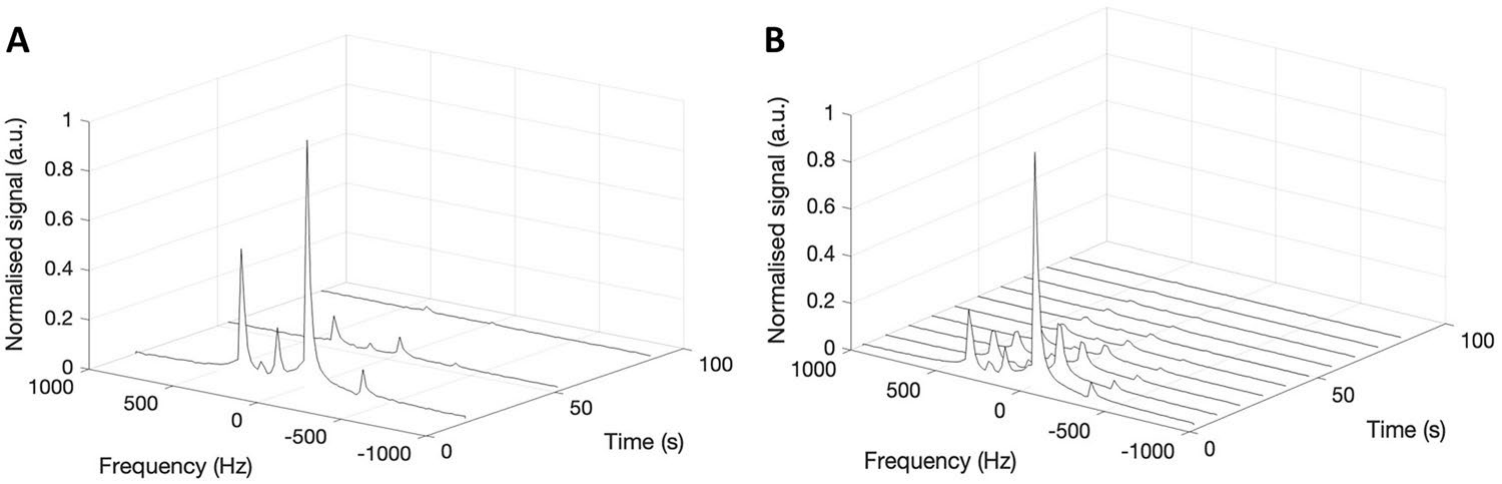
(**A**) Dynamic ^13^C spectra of the fully sampled CSI in the kidneys of a pig. (**B**) Dynamic ^13^C spectra of the under sampled CSI prior to the SENSE reconstruction in the kidneys of the same pig.

Figure 5 shows tracked metabolic dynamics in the kidneys of a different porcine subject from the previous figures. In both Fig. 5A,B, spectral peaks are well separated to capture metabolic data. The increased temporal resolution and a more detailed decay time course are achieved through the accelerated CSI acquisition as demonstrated in Fig. 5B when compared to the spectra from the standard, fully sampled, CSI acquisition in Fig. 5A.

#### Metabolic imaging

**Fig. 6:**
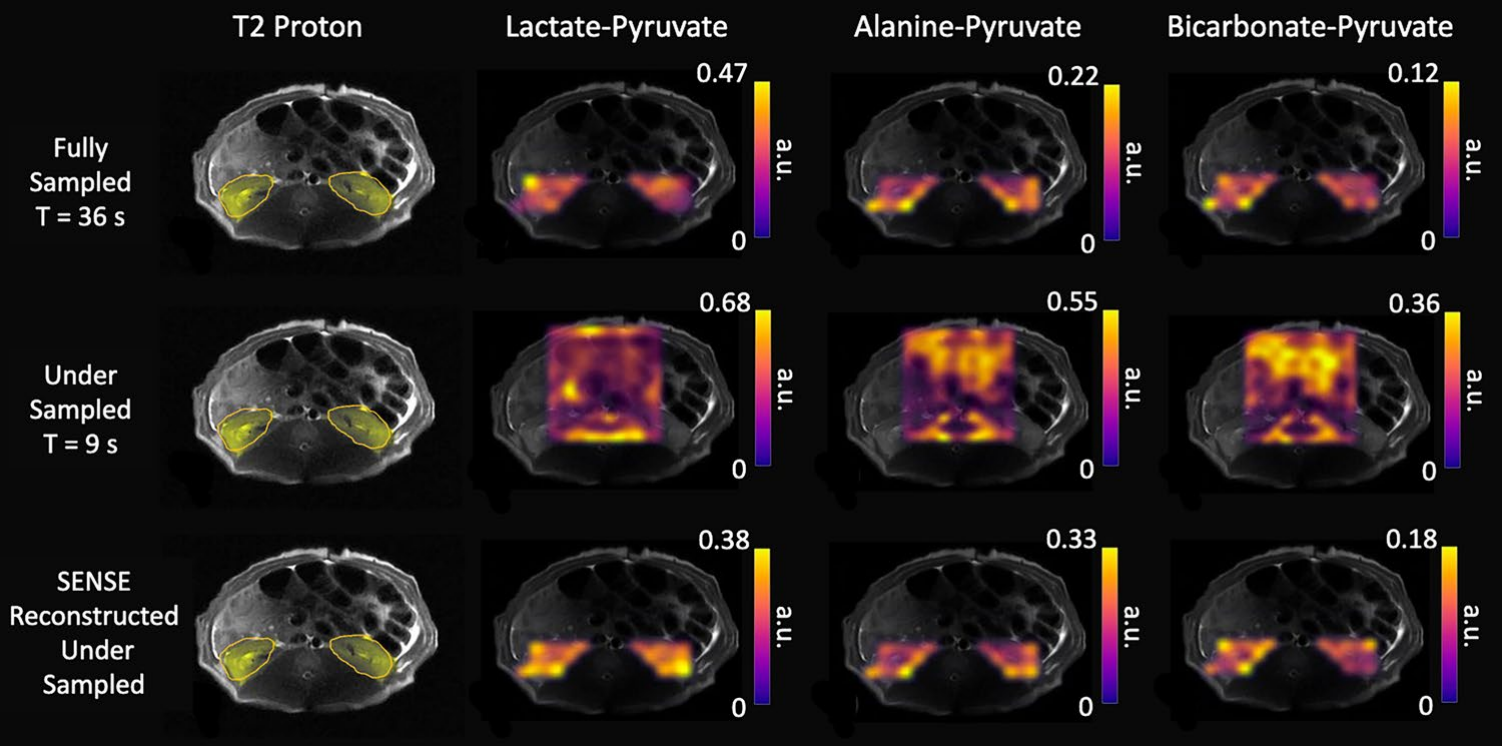
Ratio maps of lactate-to-pyruvate, alanine-to-pyruvate, and bicarbonate-to-pyruvate were calculated to show metabolism in the kidneys in the single scan fully sampled image, the under sampled image, and the SENSE reconstructed under sampled image of a healthy representative porcine subject. The axial $\textrm{T}_2$ images have also been shown to indicate the kidney locations in yellow ROIs.

In Fig. 6, ^13^C imaging demonstrates the acceleration obtained by implementing the SENSE reconstruction of the metabolite ratio distributions in the kidneys of a healthy pig. In the top row, ratio maps of lactate-to-pyruvate, alanine-to-pyruvate, and bicarbonate-to-pyruvate show values taken over the course of 36 s. The aliasing due to the reduced FOV and the acceleration down to 9 s is illustrated in the under sampled images in the second row of Fig. 6. In the bottom row, SENSE reconstructed images have recovered the folding effect at the kidneys and exhibit ratio distributions very similar to those demonstrated in the fully sampled images. The mean and standard deviation of the metabolic ratios within the kidneys from the fully sampled maps and the SENSE reconstructed maps, both summed across scans to add up to 72 s total acquisition time, were produced in three pigs as shown in Table 1. The fully sampled CSI in one of the subjects (pig 3) is omitted due to a technical issue that prevented the image from being acquired, but the mean and standard deviation values from the other two fully sampled metabolic ratio maps within the kidneys were: lactate-pyruvate (0.44 ± 0.11), alanine-pyruvate (0.19 ± 0.04), and bicarbonate-pyruvate (0.09 ± 0.01). The mean and standard deviation metabolic ratios of all three subjects in the SENSE reconstructed maps within the kidneys were: lactate-pyruvate (0.31 ± 0.02), alanine-pyruvate (0.23 ± 0.03), and bicarbonate-pyruvate (0.13 ± 0.02). The coefficients of variation of the SENSE reconstructed ratios within the kidneys for these three pigs were calculated to be: lactate-pyruvate 6.6%, alanine-pyruvate 13%, and bicarbonate-pyruvate 15%.

## Discussion

Hyperpolarized ^13^C MRI offers unparalleled potential for studying in vivo metabolism. However, its clinical translation faces challenges due to the limited lifetime of the hyperpolarized signal. To address this, various temporal acceleration strategies have emerged, each with their own advantages and limitations. This study implemented acceleration using k-space under sampling, in particular, SENSE reconstructed parallel imaging to achieve four-fold acceleration using a robust method. In current literature, the high level approaches in temporal acceleration for hyperpolarized MR imaging include: acquiring multiple k-space points per TR, under sampling during k-space traversal, decreasing TR, and harnessing spectral or spatial prior knowledge during data reconstruction. To enhance their benefits, different approaches may be combined, which is covered more extensively in the following references^22,27^.

The challenge in developing fast HP ^13^C is to balance high temporal resolution, spatial resolution, spectral resolution, and usability of the technique. Fast imaging SPSP allows for very efficient scan times that is much faster than that implemented in this study. However, SPSP relies on a known spectra with a well-separated profile to compensate for the low spectral resolution making the technique vulnerable to center frequency shift or inhomogeneous $B_0$. IDEAL has also been used with spiral trajectories, EPI and other k space under sampling methods to create very fast scans but suffers from bleed through artifacts. Compared to the method used in this study, imaging with shortened TR, such as bSSFP, is much faster at data acquisition but often has low spectral resolution and the signal is highly $\textrm{T}_1$/$\textrm{T}_2$ weighted. Fast spectroscopic methods have good spectral resolution, although inferior to CSI, and harness frequency-encodes to reduce scan time such as spiral/radial k-space trajectories and EPSI. While offering very short scan times, fast spectroscopic imaging requires the correct gradient system to be installed at the imaging site and the technique is limited by the hardware gradient slew rate in order to obtain maximum acceleration. Non-cartesian k space trajectories also produce artifacts that requires further complex post-processing to recover an interpretable image.

This study implemented acceleration using k-space under sampling of CSI in combination with SENSE reconstruction. SENSE CSI requires scan time that is longer than other ultra fast acquisition methods, but the implementation for SENSE is simple because images are accelerated by limiting the FOV. Because SENSE accelerates in image space, the acceleration artifacts are also embedded in the image domain and the reconstruction algorithm becomes a straightforward geometric problem to recover the original image. Therefore, SENSE requires a multi-channel receive coil and needs a sensitivity map in order to reconstruct the image. Although SENSE was used in this study, other parallel imaging methods can be used to accelerate in the k-space domain, such as GRAPPA and CAIPIRINHA. However, acceleration in the k-space domain requires the implementation of specialised sequences, whereas the approach taken in this study was enabled simply through the standard manufacturer supplied CSI sequence. In addition, acceleration in the k-space domain produces acceleration artifacts that are not geometrically easy to reconstruct and requires further manipulations to recover the aliasing embedded in the k space data. Compressed sensing, which implements incoherent k-space under sampling, is another method for acceleration for sparse, high resolution, high SNR data. However, this method requires complex and computationally heavy reconstruction, which lowers the usability of the technique, and the nominal resolution for ^13^C imaging is low.

Although SENSE CSI is not the fastest acquisition approach currently available, it can be used to obtain metabolic images with sufficiently high temporal resolution to monitor the metabolic timecourse whilst retaining spectral and spatial resolution in cases where carbon sensitivity profiles cannot be taken. The novel approach of using sodium sensitivity profiles with accelerated SENSE-reconstructed CSI demonstrated alias-corrected images both in phantom and in vivo pig kidney studies.

The strengths of this accelerated CSI approach were demonstrated in the in vivo experiment, where metabolic information was successfully acquired with a four-fold temporal acceleration through reducing FOV. Furthermore, the FOV used and the coil implemented in this study can be applied directly to a clinical setting, such as metabolic brain imaging, without any modifications. The use of the dual-tuned receive coil and sodium sensitivity maps eliminated the need for co-registration and additional HP injections. The high temporal resolution produced a detailed decay curve, useful for further metabolite kinetic modelling or denoising. In cases with unknown metabolite spectra or limited HP MRI specialist knowledge, this robust acceleration method ensures comprehensive capture of metabolic signals, mitigating the risk of missing spectral data. Successful SENSE reconstructed metabolic imaging, including metabolite ratio maps, were repeated in the porcine subjects to produce coefficients of variation of the SENSE reconstructed metabolic ratios within 15% across three pigs in the kidney ROIs. It is noted that there is an apparent decrease in the lactate-to-pyruvate ratio observed between the fully sampled and undersampled data, however, the small sample size and the fact that the lactate-to-pyruvate ratios for the FS CSI and US CSI are all within one standard deviation of one another makes it impossible to clarify if this is a systematic difference. Possible factors that could explain such a difference include: biological differences due to the timing of anesthesia, a biological response to the previous injection of hyperpolarized pyruvate, SNR variations, and scan variations.

The downside of this accelerated CSI approach is the need for a specialised coil and that the acceleration factor is limited by coil hardware. However, all centers undertaking hyperpolarized studies will need dedicated hardware and ^13^C coils so the limitation is minimised. Although other accelerated ^13^C imaging methods are better at achieving very high temporal resolution, such as SPSP, CSI is readily available on most major MRI systems, robust to $B_0$ fluctuations, and easy to use. For the data acquisitions presented, the spectral bandwidth and the number of acquisition points were constrained to minimise acquisition time leading to a limited spectral resolution. As such, the use of spectral fitting approaches were not appropriate for the analysis of the acquired data and trapezoidal peak integration was chosen due to the simplicity of the method for effective quantification of the spectra, which had well separated peaks, high signal-to-noise ratio (SNR) and no asymmetry. Future work to investigate the application of spectral fitting approaches may contribute to further precision in metabolite analysis. Accelerated CSI coupled with SENSE reconstruction give ^13^C images with sufficiently high spatial and spectral resolution to observe metabolism in the kidneys by implementing a simple and computationally inexpensive reconstruction. Further work will look to optimise image post-processing, signal fitting, and k-space acquisitions for increased SNR. This novel approach of using sodium sensitivity profiles with accelerated SENSE-reconstructed CSI offers a promising solution for metabolic imaging in diverse clinical scenarios.

## Methods

A Specific Anthropomorphic Mannequin (SAM) phantom filled with ethylene glycol doped with 17g/L of NaCl (291 mmoL/L ^23^Na concentration) and four female Danish domestic pigs were imaged using a 3T GE MR750 MRI scanner (GE HealthCare, WI, USA). The porcine experiments were conducted in accordance with the license and ethical review granted by the Danish animal inspectorate regulations (2019-15-0201-00298). Experiments were performed in accordance with appropriate guidelines and regulations, with appropriate clearance through relevant ethical committees, and are reported in accordance with the ARRIVE guidelines. Sodium and carbon images were acquired using a ^13^C clam-shell transmit coil (Rapid Biomedical, Germany) and an eight-channel flexible ^23^Na/^13^C receive coil (JD Coils, Germany), shown in Fig. 2. It is assumed that the commercial transmit coil has the same B1+ profiles for the ^13^C and ^23^Na frequencies. Furthermore, the receive coil is a multi-channel array coil dual tuned to sodium and carbon frequencies, and data is simultaneously acquired from all elements. The detailed performance of this exact receive coil at the sodium and carbon frequencies is quantified in the literature by Sanchez et. al^10^. Sanchez et. al have quantified sensitivity maps to be the same in phantoms between the two frequencies for the same coil used in this experiment, including the single slice sensitivity profile figure (replicated with permission in Supplementary Fig. 1). A Bloch–Siegert method^28^ was used to calibrate for the sodium and carbon center frequency and transmit gain. Free induction decay (FID) CSI, whereby a pulse sequence starts with a wide-bandwidth RF pulse, followed by phase encoding, and FID sampling, was implemented in the phantom and porcine study^29^. The image acquisition schemes in figure1 illustrate the four-fold temporal acceleration, allowing for four under sampled CSIs to be taken in a span of a single fully sampled CSI. Where multiple scans are summed, the images were added in the complex domain and the final images were created by taking the absolute values. Coil combination was carried out using the sum of squares of the signals from the separate coil channels.

### Phantom study

To assess the coil profile of the ^23^Na/^13^C receive coil (Fig. 2B) on a head-shaped phantom, signal was pre-acquired at the sodium frequency with a single-slice CSI (FOV = 240 mm × 240 mm, number of averages (NEX) = 150, flip angle (FA) = $90^{\circ }$, repetition time (TR) = 40 ms, receiver bandwidth = 5 kHz, acquisition matrix size = 24 × 24, slice thickness = 15 mm), where the FOV was larger than the phantom. The array coil was wrapped around the head phantom but was too short to wrap the whole circumference as illustrated in Fig. 2C.

A second, single-slice CSI at the carbon frequency was collected on the same set up as the sodium acquisition without alterations to the coil nor the phantom geometry. This CSI (FOV = 240 mm × 240 mm, NEX = 8, FA = $50^{\circ }$, TR = 29.3 ms, bandwidth = 50 kHz, matrix size = 24 × 24, slice thickness = 40 mm) had a FOV identical to that of the sodium acquisition.

In the post-processing steps, the coil-wise sodium sensitivity maps were produced by normalizing the sodium images to the highest signal out of all coil elements, smoothing the signal, and masking out data beyond the receive coil. Signal outside of the phantom was masked out.

To create the carbon images, each voxel spectrum was fitted with a single Lorentzian peak to one of the ethylene glycol resonances. To create an under sampled image with an acceleration factor of R = 4, the fully sampled carbon CSI k-space data was artificially reduced by filling every other row and column in the k-space matrix with zeros. This artificially under sampled image was reconstructed with a 2D SENSE algorithm in MATLAB (R2023b, Mathworks) using the sodium sensitivity maps to correct for the acceleration artifact.

To create the R = 4 g-factor map, a set of 100 noise images (R = 1) and another set of 100 accelerated noise images (R = 4) were reconstructed with a 2D SENSE algorithm. The ratio of the standard deviations of the R = 1 and R = 4 reconstructions was mapped per voxel to create the g-factor map.

### Porcine study

The pigs were sedated with zoletil mix 0.1 mL/kg IM (25 mg/mL tiletamine + 25 mg/mL zolazepam) Intravenous (i.v.) access was obtained through an ear venflon. Pigs received a 3 mL propofol (B. Braun Medical A/S) bolus through the i.v. and were intubated.

Proton imaging was acquired with the body coil using a $\textrm{T}_2$-weighted fast spin echo sequence (FOV = 360 mm × 360 mm, $\textrm{FA} = 90^{\circ }$, TR = 4300 ms, echo time = 121 ms, matrix = 512 × 512, slice thickness = 10 mm). Pigs received two injections of approximately 30 mL of 250 mM hyperpolarized pyruvate, prepared in a DNP system (SpinAligner, Polarize, Denmark) as previously described^30^, administered intravenously at approximately 5 mL/s and flushed with 20 mL saline. Imaging commenced 20 seconds after injection completion. Injections were separated by approximately 1.5 h. For the intervening time, anaesthesia was maintained with propofol (10 mL/h), midazolam (10 mL/h), and fentanyl (4 mL/h).

For sensitivity mapping, a single slice sodium CSI was acquired (FOV = 240 mm ×x 240 mm, Averages (NEX) = 16, $\textrm{FA} = 90^{\circ }$, TR = 38.5 ms, receiver bandwidth = 5 kHz, matrix = 24 × 24, slice thickness = 10 mm). Either a fully sampled or under sampled ^13^C CSI was acquired after each HP injection (fully sampled FOV = 240 mm × 240 mm, under sampled FOV = 120 mm × 120 mm, NEX = 1, $\textrm{FA} = 106^{\circ }$, TR = 64.1 ms, bandwidth = 5 kHz, fully sampled matrix = 24 × 24, under sampled matrix = 12 × 12, slice thickness = 10 mm, fully sampled temporal resolution = 36 s, under sampled temporal resolution = 9 s), where the under sampling was achieved through limiting both the FOV and the matrix size in each direction, preserving the spatial resolution. One pig used in Figs. 3 and 4 had a bandwidth = 50 kHz and spectral points = 128 and thereby captured only the pyruvate peak. To create the dynamic spectra in Fig. 5, magnitude signals in the two kidney ROIs were summed after reconstruction per time point and the plots were normalized to the respective highest pyruvate values.

Metabolite signals were extracted from the spectra using the MATLAB function *trapz*, which produced the approximate integral of the signal via the trapezoidal method with unit spacing.

Subsequent image reconstructions used sodium sensitivity and carbon images with a 2D SENSE algorithm in MATLAB (R2023b, Mathworks). In other words, spectral processing was carried out, and then the SENSE reconstruction was run. Signal maps and the g-factor map were created in the same way as in the phantom study.

Within the kidney ROIs, spectral data was summed in the magnitude domain and ratio maps of lactate:pyruvate, alanine:pyruvate, and bicarbonate:pyruvate were calculated. The mean and standard deviation of the metabolic ratios within the kidneys of the fully sampled maps and the SENSE reconstructed maps are both summed across scans to add up to 72 s total acquisition time for the same temporal resolution. Ratio maps of the under sampled images are not masked due to the aliasing. The fully sampled and SENSE reconstructed under sampled images are masked around the kidney ROIs.

**Table 1:** Mean and standard deviation of the metabolite ratios in the kidneys shown per porcine subject.

	Pig 1-fully sampled	Pig 1-SENSE reconstructed	Pig 2-fully sampled	Pig 2-SENSE reconstructed	Pig 3-SENSE reconstructed
Lactate: pyruvate	0.36 ± 0.07	0.29 ± 0.04	0.52 ± 0.16	0.32 ± 0.10	0.33 ± 0.03
Alanine: pyruvate	0.16 ± 0.04	0.21 ± 0.05	0.22 ± 0.08	0.21 ± 0.06	0.26 ± 0.05
Bicarbonate: pyruvate	0.08 ± 0.02	0.11± 0.03	0.10 ± 0.02	0.15 ± 0.04	0.13 ± 0.02

The fully sampled pig 3 has no data due to a technical issue that prevented the image from being acquired.

### Ethical approval

The porcine experiments were conducted in accordance with the license and ethical review granted by the Danish animal inspectorate regulations (2019-15-0201-00298). Experiments were performed in accordance with appropriate guidelines and regulations, with appropriate clearance through relevant ethical committees, and are reported in accordance with the ARRIVE guidelines.

## Supplementary Information

Supplementary Figures.

## Data Availability

The datasets generated and/or analysed during the current study are available from the corresponding author on reasonable request.
