# Supplementary material for: Enabling SENSE accelerated 2D CSI for hyperpolarized carbon-13 imaging
Source: Sci Rep. 2024 Sep 4;14:20591. doi: 10.1038/s41598-024-70892-8 (PMC11375102; doi:10.1038/s41598-024-70892-8)

**Supplementary figure for the article entitled "Enabling SENSE Accelerated 2D CSI For Hyperpolarized Carbon-13 Imaging"**

Supplementary figure 1: Single-slice measured coil profiles of the individual array channels for 13C and 23Na. A, Magnitude images and their relative error. B, Phase images and their absolute error (referenced to the phase of channel 2). C, 13C and 23Na noise correlation matrices. The noise covariances (normalized to the maximum variance) for 13C were: 0.75, 0.75, 0.83, 0.99, 0.80, 0.66, 0.91, 1.00. For 23Na, the (normalized) noise covariance were: 0.83, 0.65, 0.77, 0.94, 0.86, 0.60, 0.95, 1.00. Reproduced with permission from Sanchez et al. (2022). Magnetic Resonance in Medicine, 88(3), 1391-1405.

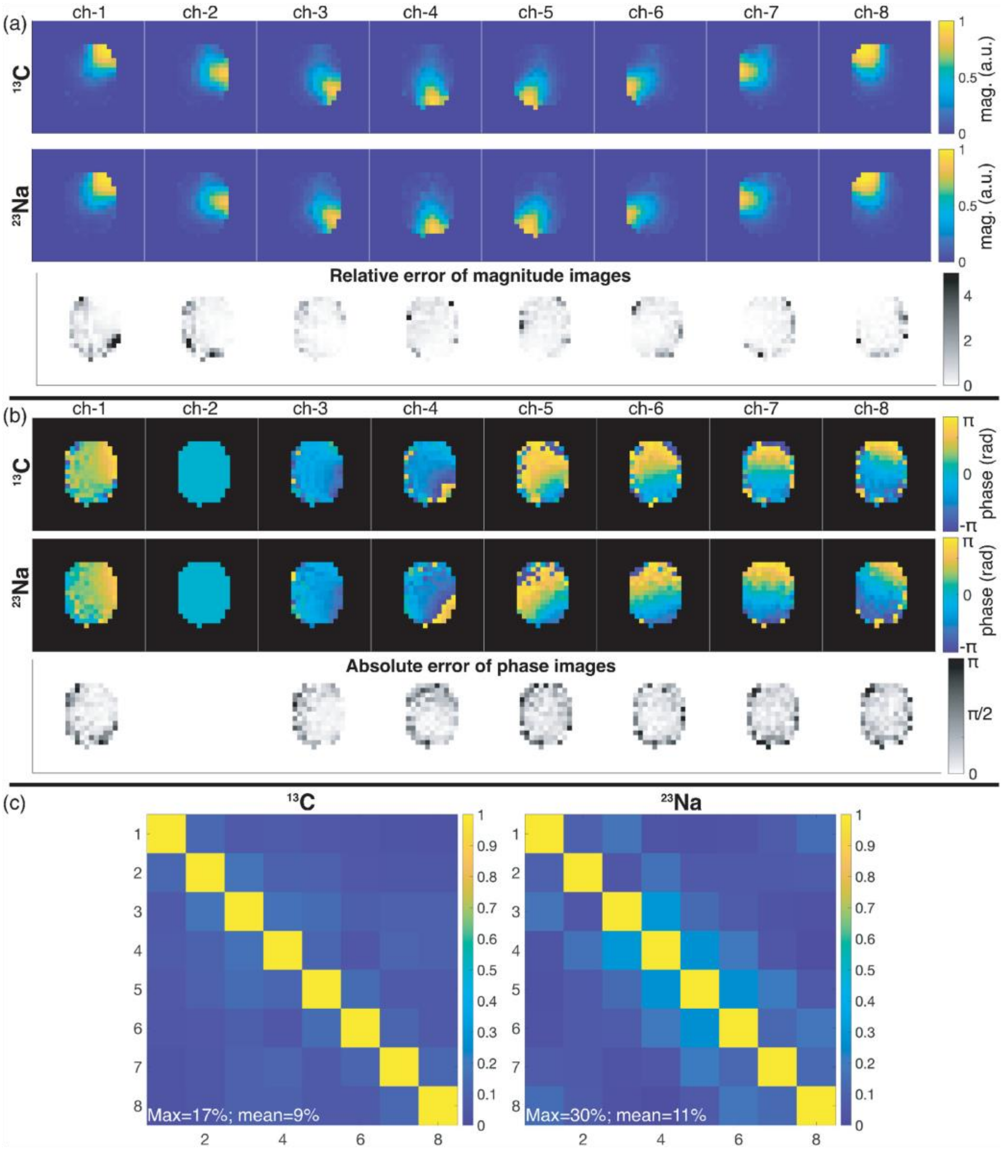

Supplement: Supplementary file 1 — Supplementary Figures. [file 41598_2024_70892_MOESM1_ESM.pdf]
